# Understanding CNS Effects of Antimicrobial Drugs Using Zebrafish Models

**DOI:** 10.3390/vetsci10020096

**Published:** 2023-01-29

**Authors:** Maria M. Kotova, David S. Galstyan, Tatiana O. Kolesnikova, Murilo S. de Abreu, Tamara G. Amstislavskaya, Tatyana Strekalova, Elena V. Petersen, Konstantin B. Yenkoyan, Konstantin A. Demin, Allan V. Kalueff

**Affiliations:** 1 Neuroscience Program, Sirius University of Science and Technology, 354340 Sochi, Russia; 2 Institute of Translational Biomedicine, St. Petersburg State University, 199034 St. Petersburg, Russia; 3 Laboratory of Preclinical Bioscreening, Granov Russian Research Center of Radiology and Surgical Technologies, Ministry of Healthcare of Russian Federation, 197758 Pesochny, Russia; 4 Neuroscience Laboratory, COBRAIN Center, Yerevan State Medical University Named after M. Heratsi, Yerevan 0025, Armenia; 5 Institute of Chemical Technology, Ural Federal University, 620002 Yekaterinburg, Russia; 6 Bioscience Institute, University of Passo Fundo, Passo Fundo 99052, Brazil; 7 Laboratory of Biopsychiatry, Scientific Research Institute of Neurosciences and Medicine, 630117 Novosibirsk, Russia; 8 Zelman School of Medicine and Psychology, Novosibirsk State University, 630091 Novosibirsk, Russia; 9 Department of Psychiatry and Neuropsychology, University of Maastricht, 6229 ER Maastricht, The Netherlands; 10 Research Institute of General Pathology and Pathophysiology, 109544 Moscow, Russia; 11 Moscow Institute of Physics and Technology, 141701 Moscow, Russia; 12 Biochemistry Department, Yerevan State Medical University Named after M. Heratsi, Yerevan 0025, Armenia; 13 Institute of Experimental Medicine, Almazov National Medical Research Centre, Ministry of Healthcare of Russian Federation, 197341 St. Petersburg, Russia

**Keywords:** antimicrobial drugs, microbiota, antibiotic, zebrafish, brain, behavior

## Abstract

**Simple Summary:**

Antimicrobial drugs, in addition to exerting antibiotic, antifungal, antiparasitic, or antiviral effects, may also affect the central nervous system and gut microbiota, thereby modulating brain and behavior. Zebrafish models can be used for studying the effects of antimicrobial drugs on the central nervous system. Here, we discuss recent findings on using zebrafish for assessing the effects of a wide range of antimicrobial drugs on brain and behavior in vivo.

**Abstract:**

Antimicrobial drugs represent a diverse group of widely utilized antibiotic, antifungal, antiparasitic and antiviral agents. Their growing use and clinical importance necessitate our improved understanding of physiological effects of antimicrobial drugs, including their potential effects on the central nervous system (CNS), at molecular, cellular, and behavioral levels. In addition, antimicrobial drugs can alter the composition of gut microbiota, and hence affect the gut–microbiota–brain axis, further modulating brain and behavioral processes. Complementing rodent studies, the zebrafish (*Danio rerio*) emerges as a powerful model system for screening various antimicrobial drugs, including probing their putative CNS effects. Here, we critically discuss recent evidence on the effects of antimicrobial drugs on brain and behavior in zebrafish, and outline future related lines of research using this aquatic model organism.

## 1. Introduction

Animal models are indispensable tools for translational biomedical research, including studying the systemic effects of various drugs in complex living systems [1]. Alongside rodent models, a small freshwater teleost fish, the zebrafish (*Danio rerio*), has become a powerful in vivo vertebrate system widely used in biomedicine [2]. Characterized by high genetic (~70%) and physiological homology to humans [3], zebrafish are also increasingly utilized in the central nervous system (CNS) research, including modeling neurodegeneration (e.g., Alzheimer’s, Parkinson’s, and Huntington’s diseases, amyotrophic lateral sclerosis) [4], epilepsy [5], affective disorders [6], addiction and various other drug-induced conditions [7]. In addition to offering multiple genetic models of CNS pathogenesis [8], zebrafish can also serve as sensitive pharmacological screens for major classes of neuroactive drugs [9], including antidepressants, anxiolytics, antipsychotics, antiepileptics, and anesthetics [10,11]. Zebrafish are also commonly used to assess central nervous action of various other chemicals, including CNS side effects of clinically used drugs [12] and neural deficits caused by toxins, environmental pollutants [13,14], and endocrine disruptors [15].

In general, antimicrobials represent a large diverse group of drugs used to prevent and treat infection, and include antibacterial (antibiotic), antiviral, antifungal, and antiparasitic agents [16]. Common *antibiotics*, classified based on their chemical structures and multiple modes of antimicrobial action, include beta-lactams, sulfonamides, aminoglycosides, tetracyclines, chloramphenicol, macrolides, glycopeptides, oxazolidinones, ansamycins, quinolones, streptogramins, and lipopeptides [17]. Typical classes of *antifungal* agents include polyenes, azoles, allylamines, echinocandins, and triterpenoids, that alter membrane permeability and/or inhibit the synthesis of the fungal wall [18]. *Antiviral* drugs have different mechanisms of action, inhibiting virus attachment, entry, uncoating, polymerase, nucleoside and nucleotide reverse transcriptase, integrase, and protease activity [19]. *Antiparasitic* drugs mainly include antiprotozoal agents [20]. A diverse array of other antimicrobial agents, acting via multiple biological mechanisms, includes chlorhexidine, triclosan, alcohols, hydrogen peroxide [21], non-steroidal anti-inflammatory drugs (NSAIDs) [22], and essential oils (e.g., basil, oregano, thyme, tea tree, coriander, and clove oils) [23].

The growing use and clinical importance of antimicrobial drugs necessitate our improved understanding of the complete spectrum of their physiological effects, including their potential effects on CNS at molecular, cellular, and behavioral levels. In addition to conventional *antimicrobial* properties that have been extensively tested in vivo and in vitro, these agents may impact CNS and behavior, both clinically and in animal models [24,25,26,27]. Like rodents, zebrafish represent a useful sensitive organism for screening the CNS effects of various antimicrobial drugs in vivo. Furthermore, antimicrobials can alter the composition of gut microbiota, and hence affect the gut–microbiota–brain axis, again modulating brain and behavioral processes. Recognizing the growing potential of zebrafish-based drug bioscreening, here we critically discuss recent evidence on central nervous effects of antimicrobial agents in zebrafish, summarize recent successes and challenges in this field, and outline future lines of research using this aquatic model organism.

## 2. Reported CNS Effects of Antimicrobial Drugs in Animal Models

### 2.1. Antibacterial Antibiotic Drugs

Mounting animal evidence demonstrates frequent CNS effects of commonly used antimicrobial drugs (also see Table 1). For example, in mice, various antibiotics, such as ampicillin, bacitracin, meropenem, neomycin, and vancomycin, evoke overt cognitive deficits, increase exploratory behavior, and alter brain expression of signaling molecules [24] and the permeability of the blood–brain barrier (BBB) [28]. Some antibiotics (e.g., ciprofloxacin, minocycline, ampicillin, neomycin, and vancomycin) lower systemic antioxidant activity [26], reduce apoptosis in rat brain [29], and increase rodent anxiety-like and impulsive behavior [30,31]. At least some of these effects may be indirect, and are probably mediated by gut microbiota status, since germ-free mice display motor hyperactivity, anxiety-like behavior [32], social deficit (e.g., social avoidance and diminished preference for social novelty) [33], as well as working memory deficits [24]. Microbiota can also affect behavioral characteristics in zebrafish, since axenic larvae exhibit hyperlocomotion corrected by microbiota colonization [34].

Triclosan, a widely used synthetic antimicrobial agent with poly-target (antibiotic and antifungal) action, also inhibits dopamine and increases acetylcholine neurotransmission, promotes neuronal apoptosis, and reduces synaptic density and axonal length in zebrafish [35]. Furthermore, triclosan downregulates the expression of brain genes that are important during neurodevelopment, including glial fibrillary acidic protein (GFAP) and myelin basic protein (MBP) that control myelination and axonal maintenance [57]. In contrast, mir-137, a short non-coding RNA associated with the mitogen-activated protein kinase (MAPK) pathway, is upregulated in zebrafish by triclosan, eventually impairing their auditory and visual sensitivity [36]. The neurotranscriptomic effects of triclosan can be mediated both by the regulation of DNA methylation [58] and by activation of other regulatory pathways, such as MAPK/ERK (extracellular signal-regulated kinases) [59].

CNS effects of β-diketone antibiotics, including fluoroquinolones and tetracyclines, have also been tested in zebrafish (Table 1). For example, a typical tetracycline antibiotic, oxytetracycline, affects the neuroendocrine system of juvenile zebrafish, such as the thyroid and adrenocorticotropic axes, as it reduces deiodinase 2 and 3, triiodothyronine T3, receptors of thyroid hormone, and whole-body cortisol levels [37]. In addition, the drug affects serotonin CNS signaling in the juvenile zebrafish, lowering brain expression of tryptophan hydroxylase, an enzyme involved in serotonin synthesis [37]. At the behavioral level, this antibiotic increases exploration and hyperactivity in zebrafish [38], whereas minocycline, another tetracycline, increased larval expression of *parkin, pink1*, and *cd-11b* genes, whose human orthologs are strongly implicated in Parkinson’s pathogenesis [39]. In line with this, minocycline evoked neuroprotective effects in a zebrafish larval model of Parkinson’s disease, preventing locomotor deficits and the loss of dopaminergic neurons [60].

**Figure 1:**
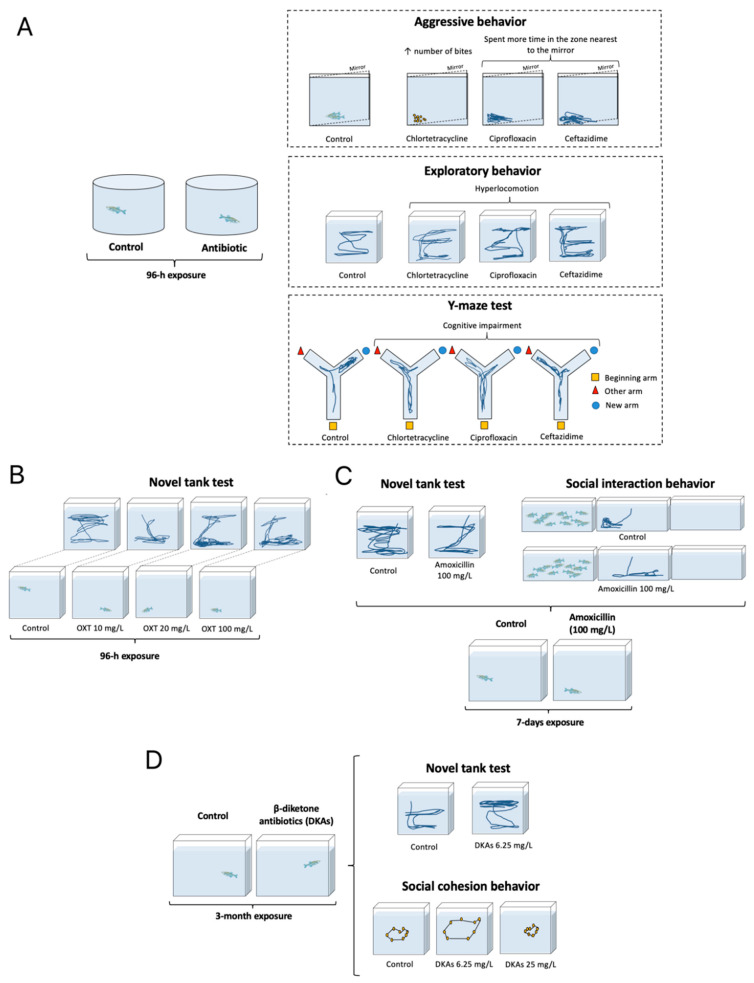
Selected examples of antimicrobial drugs’ effects on zebrafish behavior. (**A**) Exposure to chlortetracycline, ciprofloxacin, or ceftazidime for 96 h increases (↑) exploratory activity (more distance travelled), aggression (more bites in the mirror test), and cognitive deficits (more errors in the Y-maze) [42]. (**B**) Exposure to oxytetracycline (10–100 mg/L) for 96 h evokes anxiety-like behavior in the novel tank test [61]. (**C**) Exposure to amoxicillin (100 mg/L) for 7 days reduces distance travelled and social interaction [49]. (**D**) Chronic exposure to β-diketones (at 6.25 mg/L) increases time spent at the top of the test tank (an anxiolytic-like behavior) and alters (increases at 6.25, and decreases at 25 mg/L) shoaling behavior [41].

Fluoroquinols exert overt neurotoxic effects in zebrafish, impairing the development of embryos by hyperactivating the glutamate N-methyl-D-aspartate (NMDA) receptors [43]. In adult fish, exposure to these drugs increases whole-body corticotropin-releasing hormone (CRH) along with CNS levels of brain-derived neurotrophic factor (BDNF) and neuropeptide Y, but lowers plasma adrenocorticotropic hormone (ACTH) and cortisol [44]. In general, fluoroquinolones and tetracyclines are rather neuroactive in zebrafish (Table 1), and may cause ventriculomegaly, proliferation of glial cells, and neuronal apoptosis (e.g., see [40]), as well as dose-dependently increasing motor activity and altering (at low doses decreasing, and at high doses increasing) anxiety-like behavior in zebrafish [41]. Likewise, β-diketones impair zebrafish cognition (e.g., working memory) and promote aggressive behavior (Figure 1) [42].

Aminoglycosides neomycin and gentamicin damage the lateral line hair cells in zebrafish larvae, impairing locomotion and the startle response [45]. Other antibiotics have also been studied in zebrafish, including screening the CNS effects of avermectin, sulfamethoxazole, lincomycin, and amoxicillin. Interestingly, in addition to overt neurotoxicity, avermectin also increases brain expression of gamma aminobutyric acid (GABA)-A receptor in another fish species, *Carassius auratus* [62]. During zebrafish embryogenesis, sulfamethoxazole causes cerebral ischemia and brain oxidative stress, activating CNS angiogenesis, probably mediated by vascular endothelial growth factor (VEGF) signaling, since its inhibition corrects the deficits [47]. Lincomycin also has neurotoxic effects, reducing ventricular volume and neuronal numbers, but increasing systemic oxidative stress and apoptosis in zebrafish larvae, activating their acetylcholinesterase and ATPase, and decreasing locomotor activity [48]. In adult zebrafish, amoxicillin reduces locomotor and social behavior, and promotes oxidative stress in the brain, strikingly paralleling some clinical symptoms observed in autistic patients (Figure 1) [49].

Ceftazidime, a cephalosporin antibiotic, increases locomotor activity in zebrafish, impairs their learning, and promotes aggression (Figure 1) [42]. Another cephalosporin, ceftriaxone, restores normal patterns of zebrafish exploratory behavior (disrupted by ethanol withdrawal), accompanied by increased brain glutamate transport [46]. Sweroside, a secoiridoid glycoside, reduces anxiety-like behavior and improves cognitive performance in zebrafish Y-maze and novel object-recognition tests, probably due to reduced brain acetylcholinesterase activity [50], as muscarinic acetylcholine receptors are involved in both learning and memory. Furthermore, impaired cognitive status can also be explained by increased oxidative stress [63].

### 2.2. Selected Other Antimicrobial Agents

In addition to its antimicrobial effects per se, thyme (*Thymus vulgaris*) essential oil decreases anxiety-like behavior, improves cognitive function and increases acetylcholine neurotransmission in zebrafish [51]. In contrast, an antimicrobial cationic surfactant cetylpyridinium chloride negatively impacts CNS, reducing fish locomotor and social activity, also age-dependently altering neuromediators (e.g., reducing serotonin, dopamine, and acetylcholine in adults, but increasing in juvenile fish) [52].

Antimicrobial properties have also been shown for NSAIDs [22], which also exert some CNS effects in zebrafish. For example, aspirin, a typical NSAID, evokes an anxiogenic-like action in adult zebrafish, likely mediated via the serotonergic system, given its similar serotonin-modulating effect in rodents (see [53] for discussion). Exposure to high doses of aspirin markedly inhibits exploratory behavior and mobility in zebrafish, which may suggest sedative and/or toxic side-effects of this drug [54].

Another atypical antimicrobial antifungal drug, rapamycin, is an important cellular inhibitor of the mammalian target of rapamycin (mTOR) signaling [64]. Rapamycin is remarkably neurotropic in both rodents and zebrafish, reducing seizures in various spontaneous (genetic) and chemically-induced epilepsy models [55]. In addition to inactivating mTOR in zebrafish larvae with experimental epilepsy, the impairment of fine branching of GABA-ergic neurons during neurodevelopment in this model is corrected by rapamycin, suggesting some putative additional mechanisms of its CNS action beyond directly affecting the mTOR signaling [56].

Furthermore, albeit not the main scope here, mounting evidence suggests that gut microbiota may impact some CNS functions in zebrafish models, for example, during morphine addiction. Altered microbiota composition is associated with affected behavior and brain and gut gene expression in morphine-treated fish, which also show conditioned place preference (CPP) to the drug. Interestingly, these alterations are corrected by an alkaloid synenin, whereas antibiotic treatment inhibits this process, hence implicating antibiotics and gut microbiota in morphine-related behaviors in zebrafish [65]. Again, while such CNS effects are probably mediated by indirect effects of antibiotics on gut microflora, rather than by direct action on neuronal processes in vivo, this aspect clearly merits further scrutiny in future zebrafish studies.

## 3. Discussion

Antimicrobial drugs, especially antibiotics, are among the most widely prescribed and used medications, with up to almost 80% of the global population having taken them in the last 6 months [66]. Such prevalent drug usage represents a serious biomedical problem, which is further complicated by antimicrobial drug resistance and risks of multiple systemic side effects. Thus, it becomes important to better understand a fuller spectrum of physiological effects of antimicrobial agents in vivo–the task that also involves testing their CNS effects in various experimental animals (including zebrafish).

There are several research aspects to consider in regard to studying CNS effects of antimicrobials in aquatic models, such as zebrafish. For example, recent evidence of potential neuroprotective effects of some antimicrobial drugs (see above) in animal models suggests an opportunity for their use for drug repurposing. Indeed, amoxicillin reduces ischemia in mice with cranio-cerebral trauma, a neuroprotective effect associated with lower migration of T cells into the meninges [67]. Minocycline, a tetracycline antibiotic, displays its putative neuroprotective properties by reducing cerebral edema during hemorrhage, neuroinflammation, neuronal degeneration, systemic inflammation [68], and neuronal apoptosis in rodents [29]. Azithromycin, another putative neuroprotective antibiotic, reduces ischemic brain damage and restores sensorimotor function in rat pups [69], probably due to the activation of anti-inflammatory M2-type microglia [70]. Doxycycline is also neuroprotective, apparently lowering neuroinflammation by activating antioxidant enzymes in rat brains [71]. Taken together, this evidence suggests that similar effects can also be expected in zebrafish models (Table 1), and indicates that a better focus is needed on assessing potential beneficial CNS effects of antimicrobial drugs in zebrafish screenings, in addition to traditional studies assaying their unwanted side effects on the central nervous system, both in experimental models and clinically.

Can zebrafish be in principle a valid, suitable aquatic model object for assessing a wider spectrum of CNS effects of antimicrobial drugs? It seems indeed likely, since zebrafish CNS is characterized by generally conserved neuroanatomy and neurotransmission, including well-developed glutamate-, GABA-, monoamine-, acetylcholine-, histamine-, glycine- [72], purine-ergic and endocannabinoid systems [73]. Zebrafish have a complex well-developed brain, and despite the lack of cerebral cortex and a clearly defined hippocampus, show otherwise high functionality of other structures that are neurally equivalent to those of mammals [74]. Zebrafish also exhibit a wide range of well-described behaviors, allowing the study of drug effects on locomotor, anxiety- and depression-like, and social phenotypes [75,76,77]. Collectively, this enables the use of zebrafish in translational modeling of neurodegenerative, affective, psychotic, neurodevelopmental, and addictive disorders [4,78,79].

Another advantage of zebrafish models for CNS drug screening is the economic benefit of such research (relative to that in rodents), given the simplicity of fish husbandry, handling, and experimental manipulation (e.g., compare adding drugs to fish water vs. using laborious systemic injections in rodents). Furthermore, these fish are characterized by external fertilization, allowing the eggs and embryos to be easily manipulated, which, in addition to optical clarity (including some adult strains, such as *casper* zebrafish), facilitates successful application of such aquatic models in assessing drug toxicity [80].

Overall, zebrafish hold a remarkable potential for drug development. For example, Prohema, a stabilized prostaglandin E2 (PGE2) product that increases regeneration of bone marrow, has now reached clinical trials [81]. Another relevant example is the ability of cyclooxygenase inhibitors to suppress the leukemia-like phenotype, first demonstrated in zebrafish and further validated in other animal models [82,83]. It is likely that such effects can therefore also be extended to CNS processes. For instance, the beneficial action of ramipril and quinapril, angiotensin-converting enzyme inhibitors, has been demonstrated in larval zebrafish, showing therapeutic effects on intracerebral hemorrhage [84]. Neuroprotection in zebrafish is included by PROTO-1, a benzothiophenacarboxamide that counteracts ototoxic effects of neomycin on fish hair cells [85]. Screening of nearly 400 PROTO-1-like drugs has identified a stable neuroprotective compound that has reached the clinical trial phase [86]. Thus, zebrafish continue to emerge as a suitable system for evaluating potential beneficial CNS properties of antimicrobial drugs.

Studying CNS effects in zebrafish has linked specific physiological profile of antimicrobials to altered neurotransmission (e.g., the acetylcholine, dopamine, and serotonin systems), neuroendocrine signaling (e.g., modulating thyroid and corticotropic axes), and other cellular processes (e.g., decreased or increased CNS oxidative stress, altered BDNF levels, see Table 1 for details). On the one hand, such studies are important since they are mechanistic in nature, and can show which genes are up- or down-regulated by the drug in question. For example, a new pathway of regulation of the *mir-125b* gene by triclosan in zebrafish, showing increased expression of mir-125b (that can be neurotoxic) via a novel, previously unrecognized signaling pathway [59], reflects a fundamental value of this type of research in zebrafish. Furthermore, antibiotics can impact DNA methylation, and hence exert epigenetic effects in the brain [58]. Thus, analyses of the entire spectrum of effects of antimicrobial agents in zebrafish may help explore the fundamental mechanisms of regulation of CNS development and functioning.

Nevertheless, multiple questions remain open in regard to assessing CNS effects of antimicrobial drugs by experimental models in general, and by using zebrafish screens in particular (see Table 2 for a summary of selected open questions in this field). For example, albeit poorly studied, the noradrenergic system in many ways has similar pathways and targets to the serotonergic system. As the latter is often affected by various antimicrobials (Table 1), it can be interesting to assess putative drug-induced central noradrenergic effects as well. Likewise, the reported impact of antimicrobial drugs on the thyroid and corticotropic axes (Table 1) raises the possibility of indirectly affecting other components of the endocrine system, given extensive horizontal connections within this system.

Recent data show that antimicrobials can impact both neurons and glial cells. In zebrafish, while some antibiotics induce neuronal apoptosis accompanied by glia proliferation [40], the exact effects of these drugs on glial cells remain unexplored. Rodent studies demonstrate that exposure to particular antibiotics may impact gene expression in excitatory neurons, microglia, and astrocytes, with reduced efficiency of synaptic neurotransmission and cognitive deficits [88,89]. However, such effects on neurons may also be mediated by microglia. For example, antibiotics can lead to immature microglia unable to remodel dendritic spines, thereby resulting in cognitive deficits [88]. Some antibiotics, such as minocycline, inhibit microglial activation in rodents, especially the pro-inflammatory M1 microglia type [90,91]. Microglia can also induce transformation of astrocytes into the A1 phenotype, thus providing a neuroprotective effect [92]. Overall, there is likely a complicated interaction between neurons, microglia, and astrocytes, whose exact interplay in mediating CNS responses to antimicrobials is not fully understood in either rodents or fish.

Furthermore, various antibiotics modify the BBB permeability [93]. Clearly, this aspect warrants to be studied in zebrafish in a greater depth, as this would be less resource-consuming than in mammals, and may be fundamentally important, since altered BBB permeability relates to various brain diseases [94]. The link between antibiotics and neuroinflammation is another critical relevant topic for translational research, as modification of the microbiome composition can reduce neuroinflammation and alleviate Alzheimer’s symptoms [95]. However, the availability and mechanisms of this putative relationship in fish, as well as the possibility of using antibiotics to treat CNS diseases associated with neuroinflammation, merit further scrutiny.

Furthermore, antimicrobial drugs are also known to affect the epigenetic regulation of DNA expression. Indeed, some antibiotics (e.g., triclosan and minocycline) can alter DNA methylation both in rats and in zebrafish [58,90,96]. While such effects on some transcription factors (e.g., Nrf2) exist in zebrafish [59], the impact of antimicrobials on various other transcription factors, as well as at the level of acetylase and deacetylase activity, is yet to be studied in rodent or zebrafish models. An additional challenge is to explore the impact of other major experimental variables (e.g., sex, age, strain) on the brain–gut microbiota axis. For example, sex not only impacts brain pathogenesis, including autism, schizophrenia, and depression, but also affects the composition of gut microbiota [97]. Sex-dependent effects of antibiotics have been demonstrated in a mouse model of Alzheimer’s disease, affecting only males [98]. However, it remains unclear whether sex-specific CNS effects of antimicrobials can be found and replicated in fish. Likewise, the potential role of age, strain, and individual differences in CNS responses to antimicrobial drugs in zebrafish necessitates further translational studies (Table 2).

## 4. Concluding Remarks

As already noted, the aquatic zebrafish model is exceptionally well-positioned to serve as a tool for efficient and high-throughput drug screening. The latter may be highly relevant to assaying CNS effects of antimicrobial drugs. For example, unlike rodents, zebrafish larval assays allow a wide range of CNS drugs to be analyzed within minimally required time [84,99]. Importantly, zebrafish are vertebrate animals, and have a much higher genetic homology with humans [3] compared with other popular model organisms (e.g., *Drosophila* or *C. elegans)* that are commonly used for rapid drug screening. This enables the evaluation in zebrafish of a wide spectrum of physiological effects of a single drug, including assaying multiple CNS [100], cardiovascular [101], digestive [102], immune [103], and endocrine phenotypes [104].

Furthermore, the ability to perform rapid pharmacological studies enables efficient evaluation of drug–drug interactions in zebrafish, including testing combinations of a) several antimicrobial agents and b) an antimicrobial agent with another drug, that may synergistically or differentially modulate CNS functions. Moreover, it is possible to predict that studies of drug-induced CNS effects in zebrafish can be empowered by 3D modeling of their behavior, coupled by the application of artificial intelligence (AI) tools, in order to detect, recognize and decode neurophenotypic signatures for various antimicrobial drugs. The AI-based methods are becoming widely used in biomedicine, and their developing application to zebrafish drug screens clearly warrants further efforts. 

Finally, the environmental impact of antimicrobial chemicals must also be considered in the context of utilizing zebrafish models as sensitive bioscreens for potential CNS effects of such drugs. Indeed, various antimicrobial drugs are widely used by humans, contaminating wastewater and leading to their release into the environment (e.g., see data on environmental pollution by triclosan and triclocarban [105]). From this standpoint, zebrafish are particularly well-suited for assessing the environmental impact of antimicrobial agents, including their acute neurotoxicity and their long-term, delayed, and/or early developmental effects on these aquatic organisms. For example, zebrafish have already been used to evaluate the toxicity of antiviral drugs, such as lopinavir and ritonavir [106], and their utility can be extended to include screening the CNS effects of multiple other antimicrobials. The fact that fish can typically be chronically exposed to chemicals (e.g., pollutants) via water immersion also more closely recapitulates the continuous aspect of human exposure to environmental hazards (e.g., compared to a more intermittent nature of chronic systemic injections of such drugs in rodent models).

In conclusion, mounting evidence summarized here demonstrates overt CNS effects of multiple antimicrobial drugs in mammals and zebrafish, emphasizing the latter as a particularly suitable organism for evaluating potential neurotoxic side effects, and rapid screening of CNS activity, of antimicrobial drugs. As such, a rigorous unbiased search for negative and positive CNS effects of antimicrobials in zebrafish pharmacological, genetic and pharmacogenetic models will not only improve the efficiency of preclinical profiling of these drugs in vivo, but can also facilitate a better translation of these findings into rodent studies and, eventually, into clinical settings.

## Figures and Tables

**Table 1:** Selected examples of CNS effects of antimicrobials on CNS.

Classes of Drugs	Representative Drugs	Effects in Zebrafish	References
***Antibacterial***			
Phenols	triclosan	Inhibited acetylcholinesterase and dopaminergic activity, neuroapoptosis, reduced synaptic density, axonal length, higher expression of *mir-137*.	[35,36]
β-diketones	oxytetracycline	Decreased deiodinase 2/3, T3 levels, thyroid receptors, cortisol levels and serotonin synthesis. Increased exploratory behavior, motor activity, expression of *parkin, pink1* and *cd-11b*, proliferation of glial cells, ventriculomegaly, altered (decreased at low, increased at high doses) anxiety-like behavior, cognitive deficits, aggression.	[37,38,39,40,41,42]
	enrofloxatine	Increased corticotropin-releasing hormone (CRH), brain-derived neurotrophic factor (BDNF), neuropeptide Y, reduced adrenocorticotropic hormone (ACTH) and cortisol, proliferation of glial cells, ventriculomegaly, altered (decreased at low, increased at high doses) anxiety-like behavior, cognitive deficits, aggression.	[39,40,41,42,43,44]
Aminoglycosides	neomycin, gentamicin	Damaged lateral line hair cells.	[45]
Cephalosporins	ceftazidime	Increased locomotor activity, aggression and cognitive deficits.	[42]
	ceftriaxone	Corrected exploratory behavior (disrupted by ethanol withdrawal), increased glutamate uptake.	[46]
Sulfonamides	sulfamethoxazole	Cerebral ischemia, oxidative stress.	[47]
Lincosamides	lincomycin	Reduced ventricular volume, neuronal loss, locomotor activity, systemic oxidative stress and apoptosis, increased whole-body acetylcholinesterase and ATPase activity.	[48]
Penicillins	amoxicillin	Decreased locomotor activity, social behavior and oxidative stress.	[49]
Secoiridoids	sweroside	Reduced anxiety, improved cognitive performance, reduced brain acetylcholinesterase activity and oxidative stress.	[50]
***Others***			
Essential oils	extract *from* *Thymus vulgaris*	Decreased anxiety-like behavior, improved cognitive function and acetylcholine neurotransmission.	[51]
Cationic surfactants	cetylpyridinium chloride	Reduced locomotor and social activity, with lower serotonin, dopamine, and acetylcholine in brains of adult fish, but higher in juveniles.	[52]
Non-steroidal anti-inflammatory drugs (NSAIDS)	aspirin	Decreased anxiety, exploratory behavior and mobility.	[53,54]
mTOR inhibitors	rapamycin	Reduced seizures in epilepsy models.	[55,56]

**Table 2:** Selected open questions related to screening CNS effects of antimicrobial drugs in zebrafish.

Open Questions
Is it possible to repurpose antimicrobials (i.e., in order to discover novel neurotropic drugs among already clinically approved antimicrobial drugs), and how can zebrafish models facilitate this process? Do antimicrobial drugs affect other mediator systems, including noradrenaline, histamine, and opioid systems? Do these agents affect the neuroendocrine axis, and how? Can CNS diseases associated with monoamine systems be treated by modulating the microbiome with antibiotics? Can hormonal disorders be similarly corrected by altering gut microbiome with antibiotics? Does the blood–brain barrier permeability in zebrafish change following treatment with antimicrobial drugs? Can antimicrobial drugs affect epigenetic mechanisms in zebrafish brain? If yes, how do these responses correlate with those seen in rodents, and clinically? Do antimicrobial drugs have trans-generational CNS effects in zebrafish? What are potential neurogenomic effects of antimicrobials in zebrafish models? How do these responses correlate with those seen in rodents, and clinically? Are there common genes in CNS expression responses among all classes of antimicrobial agents? What are molecular mechanisms underlying potential sex differences in CNS effects of antimicrobials in zebrafish models? What are the effects of various antimicrobials on glial cells in zebrafish, rodents, and humans? Are these effects similar and consistent across taxa? To what extent are the CNS effects of rapamycin mediated via its inhibitory action on the mTOR signaling pathway in the brain? Do antimicrobial drugs affect addiction in general, besides their reported effects on morphine addiction? Traditional medicines, including Chinese and American traditional medicines, have been studied in zebrafish models exploring their CNS effects (e.g., [87]). Can these traditional medicines be used to develop novel antimicrobial drugs and agents with beneficial neurotropic profiles, and how can zebrafish screens facilitate their development? Can zebrafish models be used to assess CNS effects of topical (e.g., skin) antimicrobial drugs? Can antimicrobial drugs specifically affect the blood–brain barrier? Are these effects generally similar across taxa? Are there strain (e.g., AB vs. Tübingen fish) differences in antimicrobial drug responses and antimicrobial resistance in zebrafish models? If yes, are there strain differences in CNS responses of zebrafish to antimicrobial agents? Are there age-specific aspects of zebrafish CNS responses to antimicrobial drugs? Are there individual differences in zebrafish CNS responses to antimicrobial drugs? For example, do shy vs. bold zebrafish respond differently to the same antimicrobial drug behaviorally, or in terms of CNS biochemistry? Can artificial intelligence (AI)-based chemo-phenotypic screening and chemical modeling be used to detect and/or predict CNS effects of common antimicrobial drugs? Can these AI-based tools empower drug repurposing based on CNS screening of antimicrobial drug effects in zebrafish?

## Data Availability

Not applicable.
